# Effects of environmental factors and management practices on microclimate, winter physiology, and frost resistance in trees

**DOI:** 10.3389/fpls.2015.00259

**Published:** 2015-04-28

**Authors:** Guillaume Charrier, Jérôme Ngao, Marc Saudreau, Thierry Améglio

**Affiliations:** ^1^Department of Botany, University of Innsbruck, Innsbruck, Austria; ^2^INRA, Clermont-Ferrand, France; ^3^Clermont Université, Université Blaise Pascal, Clermont-Ferrand, France

**Keywords:** frost resistance, carbon balance, water status, stress interaction, microclimate, environmental factors, anthropogenic impact, risk assessment

## Abstract

Freezing stress is one of the most important limiting factors determining the ecological distribution and production of tree species. Assessment of frost risk is, therefore, critical for forestry, fruit production, and horticulture. Frost risk is substantial when hazard (i.e., exposure to damaging freezing temperatures) intersects with vulnerability (i.e., frost sensitivity). Based on a large number of studies on frost resistance and frost occurrence, we highlight the complex interactive roles of environmental conditions, carbohydrates, and water status in frost risk development. To supersede the classical empirical relations used to model frost hardiness, we propose an integrated ecophysiologically-based framework of frost risk assessment. This framework details the individual or interactive roles of these factors, and how they are distributed in time and space at the individual-tree level (within-crown and across organs). Based on this general framework, we are able to highlight factors by which different environmental conditions (e.g., temperature, light, flood, and drought), and management practices (pruning, thinning, girdling, sheltering, water aspersion, irrigation, and fertilization) influence frost sensitivity and frost exposure of trees.

## Introduction

Frost-related stresses strongly influence altitudinal and latitudinal plant distributions (Gusta et al., 1983; Koerner, 1998; Gansert, 2004; Larcher, 2005; Charrier et al., 2013a). Of different weather hazards, freezing events cause the greatest economic losses in agriculture (Snyder and Melo-Abreu, 2005; Snyder et al., 2005). A single freezing event can cause losses of hundreds of millions of dollars in fruit and tree production (Attaway, 1997). Most fruit species currently growing in temperate zones originate from warmer climates, especially Asia, e.g., walnut (Fornari et al., 2001), apple, pear, and plum trees. In the selective breeding process that began in the Pliocene (Manchester, 1989), high yield and strong pathogen resistance were the principal goals, rather than frost resistance (Fady et al., 2003). Although frost dramatically constrains life forms and generates enormous economic losses, it has not been as thoroughly studied as other biotic or abiotic stresses such as drought or herbivory. The main reason for this may be that damage occurs when trees seem to be inactive, and damage may become visible only in the subsequent growing season.

Frost risk (probability of frost damage) increases when hazard (probability of a given freezing temperature) coincides with vulnerability (frost sensitivity). Frost sensitivity is the converse of frost resistance. During the annual cycle of growth and dormancy, the transition periods in autumn and spring are the most risky. This is when plants are most vulnerable and there is a moderate probability of freezing. The probabilities of autumn and spring frosts are comparable (Spulak and Balcar, 2013). In autumn and spring, moderate freezing events can significantly damage trees, but during the winter, the temperatures that cause damage (i.e., frost resistance) are much lower than the temperatures that trees encounter (Larcher et al., 2010; Kollas et al., 2014).

**FIGURE 1:**
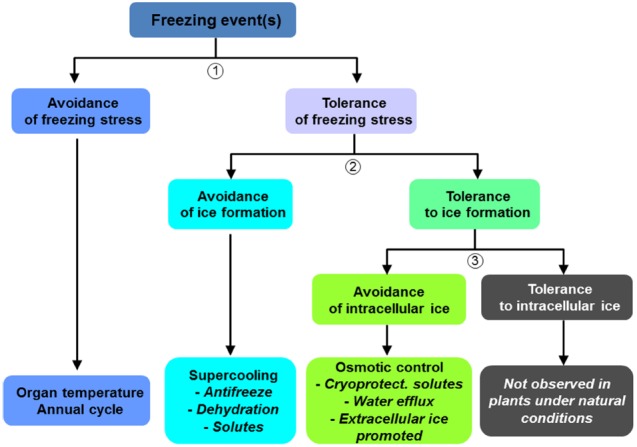
**Strategies developed by trees to avoid or tolerate freezing stress and ice formation (adapted from Levitt, 1980)**.

Phenological processes are particularly important for frost avoidance in spring and autumn (Figure 1). Phenological stages (e.g., the induction and release of endodormancy and ecodormancy; Lang et al., 1987) control the exposure of vulnerable organs to frost (e.g., flushing buds, blooming flowers, and leaves of deciduous trees). Hence, dormancy induction and release occurs simultaneously with frost acclimation and deacclimation (Palonen and Linden, 1999; Charrier et al., 2011). After growth cessation, frost acclimation and endodormancy release are both regulated by low temperatures, whereas deacclimation and ecodormancy release are subsequently controlled by warmer, mild temperatures. Temperature is perceived within the bud, both for chilling and forcing (Bonhomme et al., 2013). However, in late successional species, photoperiod may also influence dormancy release and the timing of budburst (Basler and Koerner, 2012). Photoperiod exerts its greatest effect when chilling requirements have not been fulfilled (Laube et al., 2014). The “safety margin” (calculated as the difference between the temperatures that cause damage and minimum temperatures) is usually wide enough at the end of the ecodormancy period to avoid damage to flushing buds (Lenz et al., 2013). Nevertheless, frost damage can still occur (Rodrigo, 2000; Cittadini et al., 2006). At the warmer margins of cultivated areas, trees can experience insufficient chilling, which generates erratic patterns of flushing (Balandier et al., 1993; Bonhomme et al., 1999; Marafon et al., 2011; Chuine et al., 2014; Dantec et al., 2014).

**FIGURE 2:**
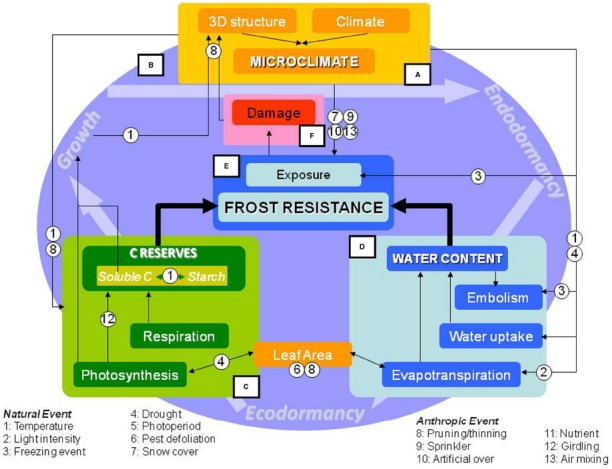
**Conceptual framework of processes involved in frost resistance development in trees.** The microclimate (Frame A) results from interactions between the climate and spatial structure of tree canopies. Trees experiencing freezing events integrate ecophysiological and internal processes throughout the entire year (Frame B). Among these biophysical factors, the balance of total non-structural carbohydrates (e.g., sugars and starch reserves; Frame C) results from various source-sink interactions during the year. Water content (Frame D), is regulated by input-output fluxes, which may lead to embolism. Non-structural carbohydrates, water content, and previous year damage affect frost resistance (Frame E) and survival (Frame F). These interactions are modulated by either natural events (numbered 1–7) or anthropogenic events (numbered 8–13).

At the individual-tree level, frost resistance is well documented. Many models are used to predict temporal changes in frost resistance (e.g., Fuchigami et al., 1982; Greer and Warrington, 1982; Leinonen, 1996; Poirier et al., 2010). However, trees are integrated organisms composed of repeated structures termed modules (Hallé et al., 1978; Kawamura, 2010). These modules (e.g., buds, shoots, branches) are histologically and spatially distinct, and located up to several meters apart. This organization results in heterogeneity in organ temperatures because of heterogeneity in microclimatic conditions (Frame A, Figure 2). At the intra-individual scale, spatial variability in frost resistance and hazard are also heterogeneous, from root system to apical buds (Charrier et al., 2013b). The consequences of frost damage for the aboveground vegetative plant parts have been less thoroughly researched than those affecting the economically important parts, such as flowers and fruits. However, the architecture of the aboveground portion of the tree influences temperature distribution (microclimate) and, therefore, potential damage. Across all plant parts, the shoot apical meristem plays a key role because temperature damage to it affects survival, ecological distributions (Nobel, 1980), and fruit production (Rodrigo, 2000). When apical buds are damaged, loss of apical dominance results in changes in growth patterns. The subsequent changes in the tree’s architecture will, therefore, modulate local environmental conditions (e.g., light, temperature, and humidity), which, in turn, can influence carbon acquisition and pest development.

The productivity and sustainability of forest and orchard trees depend on growth processes. Growth is an integrative process controlled by environmental conditions and management practices. Irrespective of the plant species or location, frost-related stresses have similar consequences, generated by low temperatures, and extracellular or intracellular freezing. Trees use similar strategies to cope with these stresses, including avoidance and tolerance. Frost tolerance usually relies on osmotic control by cryoprotectants and free water content in the tissues. Yet, empirical relationships between environmental factors (e.g., temperature and photoperiod) and frost tolerance have often been used for modeling and forecasting purposes. In this review, we propose a conceptual framework (Figure 2) that explicitly includes the ecophysiological processes underlying frost risks. We first review the different symptoms caused by chilling and freezing (see Symptoms of Low Temperature Damage), and their temporal and spatial patterns (see Modulation of Frost Resistance). We then assess the different strategies used to cope with freezing stresses (see Strategies Driving Frost Resistance). Among these, we focus on the environmental determinants and management practices that influence the osmotic control strategy (see Environmental Factors and Management Practices Influencing Frost Risk). In particular, we detail how environmental factors and management practices interact with frost resistance and within-crown microclimatic conditions. This framework could be integrated on a pluri-annual timescale with cumulative sub-lethal stresses.

## Symptoms of Low Temperature Damage

Low temperatures affect living plant cells and non-living lignified structures. Depending on whether the temperature falls below the freezing point of the sap, chilling or freezing damage occurs (Sakai and Larcher, 1987). Freezing damage also depends on the location of ice formation.

### Chilling Damage

Chilling stress, which is common in tropical species, causes (i) limited exchanges among cells due to decreased membrane fluidity and decreased activities of membrane-bound pump channels (Alves et al., 2001), (ii) decreased metabolism through decreased enzymatic activity (Lyons and Raison, 1970), (iii) protein denaturation (Siddiqui and Cavicchioli, 2006), and (iv) differential gene expression. For a review of these mechanisms, see Ruelland et al. (2009). During the leafy period, low temperatures affect the activity of photosynthetic-related enzymes. Therefore, electrons accumulate in the photosystems and generate reactive oxygen species (Ensminger et al., 2006; Mai et al., 2009; Silva-Cancino et al., 2012).

### Frost Damage to Living Cells

When temperatures drop below 0°C, water may crystallize around a nucleus, typically in the extracellular compartment (Figure 1). Latent heat released by the crystallization of extracellular (i.e., apoplastic) water can be detected as a high temperature exotherm (HTE). HTEs, recorded between –2 and –4°C, display no seasonal pattern (Pramsohler and Neuner, 2013). Tissues exhibiting secondary growth are usually tolerant to extracellular freezing, but the chemical potential of ice draws water from the intracellular to the extracellular compartment, causing cell dehydration and shrinkage (Dowgert and Steponkus, 1984). As the freezing point is lowered, cells may be able to supercool (<–10°C), or in some species, deep-supercool (<–40°C), leading to a “vitrified” cytoplasm (Wolfe and Bryant, 2001).

Whenever intracellular ice is formed, an exotherm is generated at a lower temperature than that of extracellular ice formation. Low temperature exotherms differ among species (e.g., in relation to their origin; Burke et al., 1976; Kaku and Iwaya, 1979) and among seasons (Pramsohler and Neuner, 2013). Intracellular ice results in a low water potential at the ice-water interface, which interferes with molecular bonds (hydrogen, van der Waals, and hydrophobic bonds), leading to membrane disruption and denaturation of macromolecules such as enzymes and structural proteins (Uemura et al., 2006; Ruelland et al., 2009). Under natural conditions, intracellular ice formation usually causes cell death (Wolfe and Bryant, 2001; Gusta et al., 2004; Muldrew et al., 2004).

### Frost Damage to Lignified Structures

Extracellular ice causes mechanical strain on cell walls, leading to collapse and osmotic disturbances in cells with macromolecule and membrane denaturation (Steponkus, 1981). This effect, combined with an increased volume of extracellular ice (ca. 10%), can generate frost cracks in wood (Ishida, 1963; Cinotti, 1991). Damage may also result from the biomechanical effects of freezing (Charrier et al., 2014a). Frozen sap increases wood stiffness (i.e., higher Young’s modulus, *E*), which helps crowns bear the weight of snow and ice, up to their breaking point (Umbanhowar et al., 2008). Successive freezing and thawing of apoplastic water generates water fluxes (Améglio and Cruiziat, 1992; Améglio et al., 2001). Air bubbles expelled from ice can dilate during thawing, inducing xylem embolism (Améglio et al., 1995; Lemoine et al., 1999; Hacke and Sperry, 2001; Améglio et al., 2002; Charrier et al., 2014b; Kasuga et al., 2015). A major determinant of xylem sensitivity is the diameter of the conducting elements (Davis et al., 1999; Pittermann and Sperry, 2003; Charrier et al., 2014b). After a single freeze-thaw event, embolism resulted in a 100% loss of hydraulic conductivity in *Quercus robur*, compared to 0% in *Pinus sylvestris* (Charrier et al., 2013a), However, species with narrow xylem-conducting elements can also develop embolism after many successive freeze-thaw cycles (Mayr et al., 2006, 2007). Low temperatures significantly affect the loss of hydraulic conductivity, but only in wider xylem conduits (Mayr and Sperry, 2010; Charrier et al., 2014b). Among poplar clones, trees with narrow xylem vessels were more resistant to freezing-induced embolism and grew more vigorously (Schreiber et al., 2013). Consequently, winter embolism is a major factor controlling the locations of treelines (Mayr et al., 2002, 2003, 2014; Charrier et al., 2013a). Successive freezing and thawing can also affect subsequent drought resistance in apple trees, a phenomenon known as “frost fatigue” (Christensen-Dalsgaard and Tyree, 2013, 2014).

## Modulation of Frost Resistance

Frost resistance exhibits significant spatio-temporal phenotypic plasticity. To sustain freezing temperatures, trees are able to transiently increase their frost resistance (i.e., acclimate) from a few degrees below 0°C to temperatures as low as liquid nitrogen (–196°C; Sakai and Larcher, 1987). Contrasting behaviors are also observed across organs (Charrier et al., 2013b).

### Temporal Patterns of Frost Resistance

#### Autumn

In autumn, frost acclimation is associated with growth cessation and endodormancy development (Weiser, 1970; Hänninen and Kramer, 2007; Kalcsits et al., 2009). Decreasing photoperiod and cold temperatures induce budset, growth cessation, and frost acclimation (Aronsson, 1975; Christersson, 1978; Arora and Rowland, 2011; Pagter and Williams, 2011). Frost resistance can be modeled using two independent responses, one to short photoperiods, and the other to cold temperatures (Leinonen, 1996). Similarly, two independent pathways may govern endodormancy release: one regulated by photoperiod (enhanced by warm temperatures) and the other regulated by cold temperatures (Tanino et al., 2010).

The rate of cold acclimation is related to temperature (Greer and Warrington, 1982). After a long warm autumn, a single freeze event can significantly damage apical buds and cause tree mortality (Fady et al., 2003). However, an artificially elevated temperature combined with a short photoperiod can induce significant acclimation (Schwarz, 1970; Charrier and Améglio, 2011). In that study, the authors hypothesized that low (<5°C) and warm (>15°C), but not mild temperatures, induced frost resistance because higher enzymatic activity enhanced starch hydrolysis, producing more cryoprotectant solutes (Sakai, 1966; Elle and Sauter, 2000). Cold hardening can be inhibited by an artificial increase in the water content of the tissues (Charrier and Améglio, 2011), which is efficient for low levels of frost resistance (cf. see Strategies Driving Frost Resistance). Therefore, warm soil temperatures decrease frost resistance by maintaining root system activity (Frames A, D, and E, Figure 2).

#### Winter

In deep winter, the main frost risk comes from winter drought rather than frost injury, especially at high altitudes or latitudes (Mayr et al., 2003; Man et al., 2013). When soil is frozen, dehydration of aboveground parts is not compensated by water uptake from the roots, which can be lethal to sempervirent plants (Tranquillini, 1979). In very cold areas, plants may be totally encased in ice and damaged from anoxia. Frost risks are maximal at the colder edges of plant altitudinal and latitudinal distributions (Charrier et al., 2013a). Mild spells that induce deacclimation (Kalberer et al., 2006; Pagter et al., 2011) can be damaging when frost resumes (Saarinen et al., 2011).

#### Spring

In temperate zones, spring frost risks are more abundantly documented than those for other periods. Critical damage to flowers or buds can ruin a year’s harvest (Rodrigo, 2000). In the context of climate change, many studies have focused on forecasting frost risk, but these analyses have yielded conflicting results. Frost risk is predicted to increase (Hanninen, 1991; Inouye, 2008; Augspurger, 2013) or decrease (Murray et al., 1989; Scheifinger et al., 2003; Eccel et al., 2009; Baraer et al., 2010; Bennie et al., 2010; Dai et al., 2013). A mean temperature increase, with constant variance, would be expected to lower frost exposure, even though the sensitive stages of plant development occur earlier in the spring. Nevertheless, some authors highlight a complex pattern (Linkosalo et al., 2000, 2006; Rochette et al., 2004; Gu et al., 2008), with antagonistic effects of increasing temperatures and temperature variability (Rigby and Porporato, 2008). An increase in both the mean and variance (predicted by IPCC; Field et al., 2014) implies that freezing events could still occur and damage flushing buds. In most cases, the safety margin between damaging temperatures and frost resistance (ca. 5°C) should prevent damage (Lenz et al., 2013). However, spring damage to flowers occurs more often at the cold margins of plant distributions (Cittadini et al., 2006). Late budburst may prevent spring frost damage. However, late frost damage might have strong consequences for elongating stems and developing leaves, such as poor recovery of photosynthesis, shorter growing seasons, and less production of non-structural carbohydrates (NSC; Ball et al., 1997).

#### Summer

In summer, plants are especially vulnerable, but freezing events occur only at the edges of the ecological distributions: at high altitudes (Larcher et al., 2010; Pramsohler et al., 2012; Ladinig et al., 2013; Neuner et al., 2013) and high latitudes (Christersson, 1971; Burke et al., 1976; Gorsuch and Oberhauer, 2002). In summer, frost damages only the most vulnerable and exposed organs (fruits, flowers, and buds; Hacker et al., 2011).

#### Ontogenetic Effect

Many factors can affect frost resistance, including plant height, microclimate, and soil conditions, but these are not easy to study independently. Hence, only a few studies have focused on the effect of age on frost resistance. However, seedlings and saplings are generally more sensitive than are adults. For example, significant year-to-year increases in frost resistance are observed in juvenile *Rhododendron* spp, but not in adults (Lim et al., 2014).

### Spatial Distribution of Frost Resistance

Spatial heterogeneity in frost resistance is observed across organs and tissues (Sakai and Larcher, 1987). Differential distribution of frost resistance is both qualitative (extracellular freezing or supercooling) and quantitative (acclimation). Long-term survival of plants is tied to meristem protection. Bud development is important for primary vegetative growth and reproduction, whereas cambial development is important for secondary growth. Differences in frost resistance among organs have been described in detail for only a few species: e.g., the liana, *Hedera helix* (Andergassen and Bauer, 2002), the forest trees, *Abies alba* and *Acer pseudoplatanus* (Larcher and Mair, 1968; Larcher, 1985), and the fruit tree, *Juglans regia* (Charrier et al., 2013b).

The root system survives only moderate extracellular freezing (ca. –5 to –10°C; Stattin et al., 2012), and, except for the tap root, does not exhibit a seasonal pattern (Charrier et al., 2013b). Fine roots are the most sensitive (ca. –5°C), and can be severely damaged under bare soil (Cleavitt et al., 2008). During winter, snow cover protects roots by keeping soil temperatures above freezing (Comerford et al., 2013). The trunk is the most resistant part because it is usually protected by thick bark that has a high thermal inertia (Moran et al., 2011). However, mechanical damage can be observed on trunks in autumn when they are fully hydrated. The expansion of water (sap) during freezing can cause mechanical strain to exceed cell wall rigidity and cause frost cracks (Ishida, 1963; Cinotti, 1991).

Of the different plant tissues, bark is the most resistant, much more so than wood (Arora and Wisniewski, 1994; Charrier et al., 2013b). Buds are probably the most sensitive and most frost-exposed organs (Ashworth et al., 1985; Pramsohler et al., 2012). Many studies have focused on the deep supercooling ability of plants and ice propagation in buds and wood (Itier et al., 1991; Jones et al., 2000; Hacker et al., 2011; Kuprian et al., 2014; Charrier et al., 2015). In winter, the buds are disconnected from the wood xylem, which may prevent ice from propagating into the bud (Hacker et al., 2011). In spring, the barrier between xylem and buds is removed, and ice propagation into the bud may cause severe damage (Pramsohler and Neuner, 2013), jeopardizing the entire year’s fruit production (Rodrigo, 2000; Rowland et al., 2013).

Inside the vegetative bud, the leaf primordia are the most sensitive parts, whereas procambium and pith parenchyma can survive lower temperatures (Andergassen and Bauer, 2002). In flowers, freezing stress on the pistil and ovules determines the potential survival of the seeds produced (Lardon and Triboi-Blondel, 1994). Buds in the lower part of the tree are more frost-vulnerable than those higher up (Read, 1967). Buds facing the sun are more resistant than those at the opposite side (Read, 1967).

## Strategies Driving Frost Resistance

As observed for most stresses (biotic or abiotic; Grime, 1977), tree frost resistance involves two main strategies: avoidance and tolerance (Levitt, 1980; Figure 1).

### Avoidance of Freezing

A stress avoidance strategy is exhibited by some species—the spatial and temporal distribution of sensitive organs and meristems provides protection from cold temperatures. Raunkiaer (1934) proposed a classification of plants according to the position of meristems during the dormant season. Meristems located belowground (e.g., Cryptophytes) or under cover (e.g., Chamaephytes) have less exposure to cold temperatures than do meristems above the snow cover (e.g., Phanerophytes). For temperate and boreal species, these adaptations allow freezing events to be avoided. At a larger scale, stress avoidance can be illustrated by the biogeographical distribution of a given species, reflecting adaptation to abiotic and biotic stresses. Dyschronism between sensitive phenological stages and freezing events is an avoidance strategy (e.g., leaf fall for deciduous species). Stress avoidance is, therefore, related to freezing temperature exposure, from the global to the microclimatic scale.

### Tolerance to Freezing

As stated above, damage to plant tissues depends on the quantity, location, and rate of ice formation (Figure 1). However, plants are able to partially control ice formation, lessening frost risk. Ice formation can be avoided by the production of antinucleators such as anti-freeze proteins (Pearce, 2001; Figure 1), which allow supercooling of the sap. Ice barriers can block ice propagation within sensitive tissues at different times (e.g., protection of buds in winter; Dereuddre and Gazeau, 1992; Pramsohler et al., 2012).

Other resistance mechanisms involve the inhibition of intracellular ice formation (Figure 1). Increased intracellular osmotic potential (mediated by solutes or aquaporins) is a strategy shared by different crop genera such as *Juglans* (Améglio et al., 2004; Charrier et al., 2013b), *Malus* (Pramsohler and Neuner, 2013), *Quercus*, *Fagus*, and *Betula* (Morin et al., 2007; Charrier et al., 2013a). Osmotic control maintains or stabilizes intracellular structures through the use of low molecular weight molecules such as mono- and oligosaccharides, polyols, amino acids, lipids, and macromolecules such as dehydrins (Yoshida, 1984; Khanizadeh et al., 1992; Arora and Wisniewski, 1996; Arora et al., 1997, 2004). An increase in cell wall thickness and stiffness to cope with mechanical strains, and a decrease in the degree of saturation of fatty acids to maintain membrane fluidity at low temperatures are also observed (Yoshida and Uemura, 1986; Uemura and Steponkus, 1994). Cryoprotectant molecules help cells withstand dehydration by excluding water from sensitive areas. Aquaporin expression significantly increases frost resistance by increasing cell membrane permeability (Peng et al., 2008a,b). In extreme cell dehydration, the remaining water is tightly bound to cell structures in a “vitrified state” (Wolfe and Bryant, 2001). Membrane stabilization during freeze-induced contraction and thaw-induced expansion is a key process in cell survival (Uemura et al., 2006). The rate of temperature change is, therefore, critical because plant cells may or may not have time for water to cross the plasma membrane.

Soluble carbohydrates, are involved in frost resistance of living cells and the refilling of water conduits, and can account for the ecological distributions of trees (Charra-Vaskou et al., 2012; Charrier et al., 2013a). Living cells are, thus, crucial for the maintenance of a functional hydraulic system. On the one hand, electrolyte leakage from damaged cells increases the tension in the apoplast near the vessels; this increases the vulnerability of vessels to embolism (Ball et al., 2006). On the other hand, vessel-associated cells are able to actively refill embolized vessels more easily by carbohydrate export (Améglio et al., 2001, 2002, 2004; Decourteix et al., 2006, 2008) and ATP-H^+^ pump channel activity (Alves et al., 2001, 2004). Aquaporins may also be involved in the recovery of xylem from embolism, as demonstrated in *Juglans regia* (Sakr et al., 2003).

The physical behavior of water and its interaction with different cellular components control the formation of ice within the tissues. The probability of ice nucleation depends on the volume of the compartment, the presence of nucleation sites, and the concentration of the liquid sap. High concentrations of cryoprotectants (mostly carbohydrates), combined with less intracellular water to freeze, are key to surviving very cold temperatures. Using the contents of water and soluble carbohydrates as input variables, frost resistance was accurately predicted independently of age, organ, and tissue (Frames C–E, Figure 2; Charrier et al., 2013b). For example, along a branch, gradients in water and carbohydrates are observed (Table 1). However, frost resistance exhibited no corresponding differences, owing to the antagonistic effects of water and carbohydrates. A non-linear interaction between these two variables suggests that modulation of water content drives low levels of frost resistance, whereas soluble carbohydrates are responsible for the greatest levels of frost resistance (<–20°C; Charrier et al., 2013b).

**TABLE 1:** **Frost hardiness, starch content, soluble carbohydrate content, and water content along a branch of walnut (length >1 m) in mid-autumn (October)**.

	Frost Hardiness (°C)	Starch (mg.g^–1^ DM)	Soluble carbohydrates (mg.g^–1^ DM)	Water content (g.g^–1^ DM)
Apex	–17.2 ± 0.6^a^	76.4 ± 1.4^b^	56.4 ± 6.3^b^	1.17 ± 0.05^b^
Middle	–18.3 ± 1.7^a^	50.6 ± 6.5^a^	42.2 ± 9.6^a^	0.98 ± 0.04^a^
Base	–17.6 ± 0.7^a^	62.8 ± 4.1^ab^	35.5 ± 6.3^a^	0.88 ± 0.04^a^

Values are mean ± standard error from n = 5 replicates. For each variable, branch locations with the same letter are not significantly different according to ANOVA and the LSD criterion (P < 0.05). Results are from Charrier (2011).

## Environmental Factors and Management Practices Influencing Frost Risk

Environmental factors and management practices interact with physiological variables in complex ways during periods of vegetative growth and dormancy (Frame B, Figure 2). Frost damage, as defined and described in Part 2, occurs when high hazard and low resistance are concomitant (Frames A, E, and F, Figure 2). Hazard and frost resistance are modulated spatially and temporally at the tree scale, and can be severely impacted by management practices. In addition, environmental factors fluctuate independently. Multiple stresses can occur simultaneously or successively (Niinemets, 2010), affecting either resistance or hazard. However, moderate stress can also act as a warning signal (“stress priming”), inducing physiological changes and triggering acclimation to subsequent stresses.

As stated previously, low water contents and high concentrations of NSCs enhance frost resistance (Frames C–E, Figure 2). Thus, heterogeneity in frost resistance may lead to heterogeneity in NSC and water contents at the end of the growing season. Such spatial variability results from the concurrent interactions and feedback between the local microclimate and tree functioning.

### Air and Organ Temperatures

#### Organ Temperature

Air temperatures are influenced by microclimate, topography, and soil characteristics (Ball et al., 1997; Blennow, 1998; Blennow and Persson, 1998). Therefore, organ temperature is driven by the surrounding microclimate, but is also modulated by the organ’s physiological state (Chelle, 2005). The within-crown microclimate is highly variable spatiotemporally, and changes according to plant architecture, structure of the surrounding vegetation, climate, and pedoclimate (Frame A, Figure 2). Thus, many factors influence organ temperature and subsequent hazards. These factors include crown characteristics (e.g., structure, size, geometry), organ characteristics (e.g., horizontal or erect orientation, height above the ground), soil properties (e.g., moisture content, color, presence of scarified ground or grass), weather conditions (e.g., wind speed, air temperature, and radiation), and the presence of cover (Leuning, 1988; Leuning and Cremer, 1988; Jordan and Smith, 1994; Ball et al., 1997; Blennow, 1998; Blennow and Persson, 1998; Mayr et al., 2006; Winkel et al., 2009). The dynamics of organ temperature have been less thoroughly studied than those of air temperature. The temperatures of leaves, fruits, and stems can be simulated by solving an energy balance equation (Sinoquet et al., 2001; Potter and Andresen, 2002; Saudreau et al., 2007), and the risk of freezing can be accurately estimated (Cellier, 1984, 1993; Leuning, 1988; Jordan and Smith, 1994).

#### Air Temperatures During a Freezing Event

Under natural conditions, two kinds of freezing events can be observed: advective frosts (intrusion of cold air) and radiative frosts (negative heat energy balance). Freezing events that damage plants are more often radiative than advective. For example, 90% of freezing events in Swedish mountainous areas are caused by radiative freezing (Lindkvist et al., 2000). Radiative frosts are comparable to dew formation: nighttime radiant cooling of an exposed organ lowers its temperature, and this may fall cause the freezing of water droplets on the organ’s surface (Monteith and Unsworth, 1990). Radiative frosts typically occur after sunset when the sky is clear and wind speeds are low. When no solar radiation is entering the system, and long-wave infrared radiation is being dissipated to the sky, the heat energy balance becomes negative. With a negative energy balance, the temperature of the system (either the air layer around the plant or the plant itself) falls (Snyder and Melo-Abreu, 2005). This occurs more frequently in narrow valleys than in concave or flat locations, and less frequently in elevated and convex areas more exposed to wind (Lindkvist et al., 2000). Thus, high winds narrow the differences between the organ and air temperatures by reducing the thickness of the boundary layer (Jordan and Smith, 1994; Michaletz and Johnson, 2006). Air temperatures are lower than soil temperatures (Friedland et al., 2003), with the lowest air temperatures being close to the ground (Leuning and Cremer, 1988; Jordan and Smith, 1994; Blennow, 1998; Battany, 2012) or in the upper canopy (Winkel et al., 2009).

#### Organ Temperatures During a Freezing Event

Canopy structure can either decrease or increase the probability and intensity of freezing. This is modulated through the structural arrangement of foliage, including canopy height, length, density, porosity, and leaf area index (Landsberg and Thom, 1971; Grant, 1984; Winkel et al., 2009), and through individual organ characteristics such as size, geometry, and orientation of the leaf or bud. These factors significantly influence intra-crown air temperatures around the organs (Michaletz and Johnson, 2006) and the exposure of organs to wind and radiation (Wilson and Flesch, 1999; Kuhrt et al., 2006; Batzer et al., 2008; Saudreau et al., 2009). A dense tree canopy buffers sensitive tissues from adverse weather conditions, reducing the probability and intensity of freezing events (radiative or advective). Minimum leaf temperatures generally remain higher in dense stands than in open stands (Blennow, 1998; Kuhrt et al., 2006; Winkel et al., 2009) because a dense canopy impedes the upward dissipation of heat and reduces the cooling effects of wind (Nobel, 1974; Landsberg et al., 1975; Phillips et al., 1983; Hamer, 1985). Under canopies, daily differences in organ temperatures are lower, and soil and air moistures are higher (Chen et al., 1993; Carlson and Groot, 1997; Wilson and Flesch, 1999; Kuhrt et al., 2006). Conversely, more open canopies increase the loss of infrared radiation when skies are clear and winds are low (Wilson and Flesch, 1999; Batzer et al., 2008; Richard et al., 2013).

At the leaf scale, larger horizontal leaves lose heat faster and become colder than small erect leaves (Jordan and Smith, 1994). Also, horizontal leaves may bear water droplets that act as nucleating agents for ice formation (Wisniewski et al., 2002; Gusta et al., 2004). Therefore, leaf wettability is a critical factor that decreases across plant species along an altitudinal gradient (Aryal and Neuner, 2010). In leaves, ice propagates faster near the leaf tip and margins than at the petiole and midvein (Ball et al., 2002). Spatial patterns of ice formation can affect the distribution and extent of damage in autumn when the duration of freezing temperatures may be too short for the whole leaf to freeze before sunrise.

The temperature of the shoot apical meristem may deviate from the air temperature by ± 4°C (Yamada and Takahashi, 2004). Phenological stage and surface wetness significantly influence the degree of supercooling that buds can undergo before freezing (Itier et al., 1993). Interspecific differences are observed depending on bud structure (e.g., thickness of the cuticle and thermal insulation provided by bristles), and these interact with environmental variables such as air temperature, vapor pressure deficit, radiation, and wind speed (Savvides et al., 2013).

Wide variability in temperature dynamics between plant parts that are exposed and unexposed to direct solar radiation has been observed on perennial woody parts such as stems or trunks (Derby and Gates, 1966; Potter and Andresen, 2002; Kuhrt et al., 2006; Mayr et al., 2006). Hence, the portions of the trunk and branches that face the sun and wind often experiences two times more freeze-thaw cycles with faster freezing and thawing compared to the other sides of the tree (Derby and Gates, 1966; Mayr et al., 2006).

#### Artificial Modulation of Frost Exposure

Artificial techniques can protect plants from radiative frosts. For instance, irrigation of plants by sprinkling them with cool water can prevent frost damage to flowers by delaying budburst (Lakatos et al., 2011). Wind machines have been used to prevent freezing by mixing air layers (Ribeiro et al., 2006). Upward facing wind machines are significantly more efficient (Battany, 2012). Using the exothermal heat of ice crystallization, water aspersion warms plant tissues by a few degrees, and this can prevent damage. By combining covers and sprinklers, organ temperatures can remain 2 to 10°C higher than air temperatures, even with temperatures as low as –20°C (Cary, 1974). Soil irrigation increases heat transfer and storage. However, wet soils, which are darker than dry soils, can increase absorption of solar radiation. Artificial shelters are relatively efficient for reducing wind speeds, but inefficient for raising minimum temperatures under wet conditions (McAneney et al., 1990). Advective frosts can be minimized by limiting cold air intrusion. Sheltering and windbreaks reduce snow blowing, slightly increasing temperatures and reducing evaporation (see Brandle et al., 2004).

Removal of biomass via pruning, thinning, and girdling, or by defoliation, significantly impacts orchard and forest trees. “Pruning” usually means removing woody parts from the canopy, whereas “thinning” usually means removing either individual or clusters of fruits or flowers. In the short-term, modification of canopy structure impacts the microclimate by increasing the energy lost by long-wave radiation (Willaume et al., 2004; Stephan et al., 2008; Frame A, Figure 2). Although canopy modification may lower the minimum temperatures during freezing events, removing lower buds can also help avoid frost exposure on the remaining buds in the orchards. The remaining buds, flowers, and fruits are located higher in the tree where the temperatures are warmer. Pruning also indirectly enhances height growth by increasing light transmittance within the canopy (Usenik et al., 2008; Weber et al., 2011; Haouari et al., 2013). This decreases the probability of frost exposure to the newly formed apical buds. This effect can be observed when pruning removes more than 30% of total twig length (Fumey et al., 2011; Forrester, 2013). In cold climates, deciduous trees should not be pruned during the dormant season. For example, see Snyder et al. (2005) for a review of problems that may occur when trees are pruned during dormancy. In grapevines, pruning in the dormant season may delay budburst and blooming, thereby reducing frost damage to the flowers (Friend et al., 2011) and delaying leaf fall (Weber et al., 2011). When defoliation breaks paradormancy, trees refoliate, exposing sensitive organs to early frosts and depleting their reserves (Wargo, 1972; Wargo et al., 1972). Following defoliation stress, budburst may occur earlier the subsequent spring, increasing frost risk (Carroll and Quiring, 2003).

### Carbon Balance

Frost resistance during winter is related to the content of NSC (Palonen and Buszard, 1997). More particularly, maximum frost resistance is correlated with total NSC in the autumn (Morin et al., 2007; Poirier et al., 2010; Charrier et al., 2013a). Thus, autumnal NSC levels are a critical variable for the coming winter—it is an integrator of the carbon fluxes and the allocation that occurs during the previous growing season. Furthermore, it may be indirectly impacted by the factors influencing carbon assimilation, respiration, and allocation (Frame C, Figure 2). Among these factors, natural or artificial biomass disturbances affect the activity of the main source and sink organs. These source and sink organs include leaves, foliar and floral buds, growing fruits, and shoots. Their impact on frost resistance may be variable, and are detailed below.

#### Winter NSC Dynamics

Winter carbon balance, as measured by NSC dynamics, is mainly influenced by the respiration rate (Ögren, 2000). Respiration is temperature-dependent, and exhibits annual changes, such as variation in the basal respiration of leaves during the year (Wythers et al., 2005; Tjoelker et al., 2009). However, very few data are available for fruit tree species (Wibbe et al., 1994).

Warm autumn temperatures delay root dormancy. Root dormancy is not induced by short days and roots have no chilling requirement to resume growth (Coutts et al., 1990; Nicoll and Coutts, 1998). Therefore, mild winters may enhance respiratory losses, which accelerate the draw-down of carbohydrate reserves (Crawford and Palin, 1981). This may lead to increased mortality of roots and the aerial parts of the plant (Fitter et al., 1998, 1999). Concurrently, the dynamic equilibrium between starch and soluble carbohydrates varies with temperature. Starch is converted into soluble sugars by amylase at both low (Sauter and van Cleve, 1991; Witt and Sauter, 1994) and warm temperatures (Sakai, 1966). This explains why NSC contents are often related to frost resistance (Coleman et al., 1992; Palonen and Buszard, 1997; Wong et al., 2003, 2009; Turhan and Ergin, 2012; Ito et al., 2013). Also, starch hydrolysis and starch-derived sugar respiration are enhanced by warmer temperatures (e.g., temperatures above 15°C). This leads to decreased starch reserves, but frost resistance may not be affected (Schwarz, 1970; Charrier and Améglio, 2011). Active transport of sugars is observed at cold, but not freezing temperatures, and sugar transport is probably involved in the resorption of air bubbles in embolized xylem (Améglio et al., 2004, 2005; Decourteix et al., 2008).

#### Modulation of Maximum NSC During the Growing Season

In autumn, NSC content is driven by the tree carbon balance. That is, the total assimilated carbon allocated to growth (e.g., vegetative, flower, and fruit), reserve compounds (mostly starch distributed among the different tree parts), and respiration. Silpi et al. (2007) and other authors showed that NSC reserves formed an active sink, driving a large part of the assimilated carbon into NSC with a wide margin between reserves and structural carbohydrates (Sala et al., 2012). This contradicts the passive pool concept that is still included in many carbon-based models (Cannell and Dewar, 1994; Lacointe, 2000; Le Roux et al., 2001; Genard et al., 2008; Franklin et al., 2012).

Maximal NSC level is also influenced by the spatial heterogeneity of the tree crown and the translocation resulting from the activities of sinks and sources. Within canopies, the branch autonomy theory suggests that little carbon (and NSC) translocation occurs (Sprugel et al., 1991). This theory partially explains why branch growth and survival are affected by local light conditions and potential source/sink imbalances. The relative autonomy among shoots or branches was mostly observed in mature trees of various fruit species (Yamamoto et al., 1999; Volpe et al., 2008; Poirier et al., 2010). In addition, recent studies using whole-canopy stable isotope labeling showed rapid, seasonally variable allocation of recently assimilated carbon to belowground parts (see the review of Epron et al., 2012). These studies highlight the need to (i) enlarge the concept of source-sink activity at the whole plant scale and (ii) integrate transport resistance mechanisms into plant models (Minchin and Lacointe, 2005).

The combination of spatial heterogeneity (resulting from tree geometry and source/sink balances) and sink activity of the NSC reserves leads to high variability in NSC location (Bory et al., 1991; Haddad et al., 1992). This inherent variability may affect winter frost resistance. Under limiting conditions (e.g., low temperatures), trade-offs between limited growth and allocation to NSC reserves should ensure sufficient frost resistance (Hoch and Koerner, 2009, 2012; Wiley et al., 2013).

#### Artificial Modulation of Source-Sink Relationships

Most studies on biomass removal have shown alterations in the source-sink relationships, but with different outcomes on carbon balance and frost resistance. Pruning influences NSC, especially starch content, and this impacts (i) the differentiation of bud meristems (Andales et al., 2006; Bangerth, 2006; Frame C, Figure 2) and (ii) dormancy induction and release (Frame B, Figure 2). By removing sinks (e.g., fruits and starches) and sources (e.g., leaves), pruning has a large effect on NSC. The effects of pruning depend greatly on the timing (summer vs. winter), although very few studies are available. Summer pruning may have no effect on NSC (Usenik et al., 2008), or may promote NSC in different aboveground organs (Den and Son, 2000; Demirtas et al., 2010). However, summer and winter pruning have positive synergistic effects on starch content the second year after treatment (Den and Son, 2000), especially after fruit harvest (Demirtas et al., 2010). Changes in pruning frequency result in modifications in NSC distribution and seasonal patterns in ornamental species. With regular pruning (e.g., annual winter pruning), local starch concentrations doubled (Bory et al., 1991). However, with irregular pruning (e.g., every 2–3 winters) higher starch concentrations were observed at the bases of side branches (Clair-Maczulatjis and Bory, 1988; Haddad et al., 1992). Much more information is needed on NSC content in response to pruning, especially in different organs, and its implications for frost resistance of these organs.

Thinning of fruits or flowers aims to manipulate the carbon sink strength and, thus, the optimization of crop load and/or fruit quality (Gordon and Dejong, 2007; Haouari et al., 2013; Mirás-Avalos et al., 2013; Frame C, Figure 2). Fruit thinning seems to (i) stimulate shoot growth (Pretorius et al., 2004; Gordon and Dejong, 2007; Forrester et al., 2013) and (ii) inhibit net CO_2_ assimilation by end-product inhibition (Francesconi et al., 1996; Wünsche et al., 2005; Haouari et al., 2013). Between these two contrasting possibilities, we hypothesize about the central role of NSC. Fruit removal may allow NSC resources to be allocated to the remaining fruits, or to other active sinks such as growing tissues. Haouari et al. (2013) found increased starch content in leaves and soluble sugars in fruits after 75% fruit thinning. Thus, thinning may compensate for lowered NSC content after drought stress (Lopez et al., 2007; Mirás-Avalos et al., 2013). Ultimately, thinning may influence the role of NSC in winter frost resistance.

Girdling, which is the partial or total removal of a ring of phloem around the bole, stops the basipetal movement of assimilates through the phloem, resulting in carbohydrate accumulation above the girdle (Mataa et al., 1998; Urban et al., 2004; Frame C, Figure 2). This practice is used to manipulate (i) tree growth and development and (ii) fruit growth, load, quality, and maturation in fruit species (Krezdorn, 1960; Barry and Veldman, 1997; Goren et al., 2004; McQueen et al., 2004; Pretorius et al., 2004; Urban et al., 2004; Boyd and Barnett, 2011). Girdled branches exhibit lower water contents, higher soluble carbohydrates, and more vigorous growth (Manoury-Danger et al., 2010; Poirier et al., 2010). Therefore, the shoots produced are more frost-resistant (Poirier et al., 2010). Below the girdled area, plants deplete their reserves for frost protection (Regier et al., 2010).

Abiotic and biotic stresses alter the processes described above. In their review, Sala et al. (2012) indicated that the negative effects of drought on carbon balance do not occur until the carbon reserves have begun to decline. This is especially pronounced when tree responses to drought are less limited by carbon availability than by accessibility and transport. Consequently, summer drought can significantly alter the levels of reserves, which may affect frost resistance the following winter (Poirier et al., 2010; Galvez et al., 2013). Very strong drought may lead to partial or total defoliation, limiting carbon acquisition. The consequences of defoliation by drought or pests appear to depend on the intensity of defoliation. When defoliation is moderate, carbon allocation to reserves can still be observed (Wiley et al., 2013). When defoliation is strong (e.g., loss of all leaves), NSC reserves are depleted (Poyatos et al., 2013; Rosas et al., 2013; Wiley et al., 2013). Plants with high levels of NSC are more likely to survive phytophagous defoliation (Wargo, 1979; Canham et al., 1999). Total defoliation late in the growing season (i.e., after growth cessation), does not significantly affect carbon balance (Morin et al., 2007; Poirier et al., 2010), but may strongly affect water balance, and this may disturb the first stage of cold acclimation (Charrier and Améglio, 2011).

### Water Balance

Water balance is altered during the winter. Water uptake is limited by the decrease in root cell membrane fluidity caused by low soil temperatures (Wan et al., 2001; Lee et al., 2012). Artificially warmed soils (>8°C) have greater root activity, leading to rehydration of aboveground organs (Charrier and Améglio, 2011). Under normal conditions, aboveground parts evaporate water through the bark. Up to half of the water content may evaporate in mid-winter (Charrier et al., 2013b). The amount of evaporated water depends on the thickness of the bark, on the microclimate (evapotranspiration ETP), and on the ratio of canopy volume to surface area. Therefore, microclimatic conditions can influence within-crown water content. Accurate prediction of water stored in the bark is possible using soil water potential and vapor pressure deficit as input variables (Zweifel et al., 2005). Under extreme conditions (e.g., frozen soils), substantial embolism can occur in the xylem, and this may result in winter drought (Mayr and Charra-Vaskou, 2007; Mayr et al., 2014).

Water availability affects plant physiology by altering the heat energy balance (Monteith, 1972), carbon balance (via stomatal closure), phenology (especially during cell expansion), and water status of plants (e.g., xylem embolism). Therefore, both drought and flooding can affect frost resistance (Frame D, Figure 2).

Although water excess and deficit have a direct effect on microclimatic conditions, it is unclear whether they affect phenology and the length of the growing season. Droughts are exacerbated by warm temperatures. However, using rain exclusion, Swidrak et al. (2013) observed that water availability had only a minor impact on the onset of growth in aboveground organs. Although locally stored water is preferentially used, drought may cause premature growth cessation. However, delayed budburst in water-limited environments may decrease the overall water demand, allowing trees to have a longer growing season (Linares et al., 2012).

Flooding is mostly observed during the dormant season from autumn to spring. With flooding, temperatures decline, but are maintained above the freezing point. Whereas aboveground parts are relatively unaffected, roots may experience hypoxia (and in extreme cases anoxia), low nutrient availability, and low soil temperatures (van Cleve et al., 1981; Lieffers and Rothwell, 1986). Fine roots are sensitive to prolonged flooding, and growth is reduced when oxygen concentrations drop below 2.0 ppm (Zinkan et al., 1974). Damaged roots exhibit reduced hydraulic conductivity, which can increase water stress and xylem injury in the subsequent summer (Lamhamedi and Bernier, 1994). Flooded roots also exhibit carbohydrate depletion, making them more vulnerable to post-anoxic injuries when water levels drop again in spring (Smith, 1994; Crawford, 1997, 2000). Therefore, successive flooding can deplete carbohydrate reserves, increase root mortality, increase drought stress, reduce anchorage, and lower resistance to wind.

Genes involved in the modulation of frost resistance are linked to dehydration-related genes (Swindell, 2006). In the short term, artificial dehydration and drought stress affect water balance and increase frost resistance (Li and Weiser, 1971; Medeiros and Pockman, 2010). However, stomatal closure has a marked effect on carbon balance (Lopez et al., 2007). The width of the safety margin between the water potentials that lead to growth cessation (limiting sink force) and those that lead to stomatal closure and cessation of photosynthesis is, consequently, a critical factor that controls the levels of carbon reserves at the end of the growing season (Mitchell et al., 2014). Frost resistance during the subsequent winter is also affected by extreme summer drought and summer warming (Kreyling et al., 2012, 2014). Prediction of forest tree dieback is related to frost risk. For example, frost may damage roots when the durations of snow cover are shortened, and this is exacerbated by summer drought (Auclair et al., 2010).

### Nutrients

Nutrients have complex effects depending on which nutrient is being supplied and when. The relationship between specific nutrients and frost resistance is unclear. Fertilization generally increases growth by increasing leaf area, light interception, and transpiration. Therefore, carbon allocation is shifted to the aboveground plant parts, but the NSC pool is reduced, leaf coloration is delayed, and budburst is advanced (Thomas and Ahlers, 1999; Nikula et al., 2011; Forrester, 2013). In droughty soils, nutrients exhibit less mobility and assimilation, and this may lead to nutrient deficiency (Chapin, 1991).

Local increases in soil nitrogen induce root proliferation in these patches. The shape of the leaves directly connected to these patches by the xylem vessels may be affected in *Acer* spp, but not in *Betula* spp (Gloser et al., 2007). This heterogeneity may subsequently affect carbon assimilation under heterogeneous light, but probably only when soil water is limiting (Thorn and Orians, 2011).

Fertilized trees are more vulnerable to frost damage, especially the meristems. Application of nitrogen fertilizer in late summer or early autumn decreases frost resistance (Thomas and Blank, 1996). High nitrogen content affects potential defoliation (Albrectsen et al., 2004) and timing of frost hardening (Nikula et al., 2011). However, nitrogen is critical for root system activity and for inducing root pressures that help the resorption of air bubbles and alleviation of embolism (Ewers et al., 2001; Gloser et al., 2007; Thitithanakul et al., 2012). Phosphorus, which is involved in cell division, is important for recovery of tissues after freezing damage. Potassium has a favorable effect on water regulation and photosynthesis in plants, but its effect on frost protection is unclear (Snyder et al., 2005). Further research is needed, especially on the potential interactions between different nutrients and frost resistance.

## Conclusion and Perspectives

The review of the different temporal and spatial patterns of frost resistance highlights two main points. First, frost damage may develop in living cells or hydraulic conducting systems with potential feedback between different levels. For example, living cells actively refill hydraulic conducting elements. Second, factors affecting exposure to low temperature, carbohydrate content, and water status are involved in frost risk. We propose that these latter factors form a foundation of an ecophysiologically-based framework of frost risk assessment. Furthermore, microclimatic conditions may modulate carbon balance, water status, phenology, and temperature itself.

According to this framework, frost resistance results from variations in carbon and water balance that occur at various time scales. Changes in carbon and water balance result from changes in microclimate during the growing season, and from management practices. Therefore, the probability of frost damage increases when resistance is low and the probability of frost exposure is high. For example, under current climatic conditions, frost risks are greatest for buds in autumn or spring, and for stems in autumn when acclimation is delayed.

The development of this framework, which describes how frost resistance is affected by environmental factors and management practices, is an important step to assess frost risk in current and forecasted future climates. Understanding the physiological processes driving frost resistance will provide more robust predictions than simply understanding the empirical relationships between environmental conditions and frost risk. However, many aspects are still unclear, especially regarding the potential interactions between the environment and physiology. Sublethal stresses may not cause mortality by themselves, but successive stressing events may progressively weaken the trees by decreasing NSC content, and this may improve the reproductive success of the most stress resistant individuals. This conceptual framework opens up new directions for further research on a pluri-annual timescale, which could be broadened to cover different species, genotypes, and locations.

## Author Contributions

GC did the drafting, with input from the other three authors.

### Conflict of Interest Statement

The authors declare that the research was conducted in the absence of any commercial or financial relationships that could be construed as a potential conflict of interest.

## Terminology

*Ecodormancy*: Temporary suspension of meristem activity controlled by environmental factors, i.e., temperature and photoperiod.
*Endodormancy*: Temporary suspension of meristem activity controlled by intrinsic factors, and released by chilling exposure, before the ecodormancy stage.
*Exotherm*: Latent heat(334J.g^−1^) released during the transition from liquid water to solid state: crystalline structure of ice being more stable than liquid water. The exotherm increases the temperature of the freezing organ. Apoplastic water freezes at higher temperatures (high temperature exotherm) than intracellular water (low temperature exotherm).
*Frost risk*: Risk of frost damage is incurred when hazard (freezing event) meets vulnerability (frost sensitivity).
*Girdling*: Partial or total removal of phloem ring.
*Hardening*: Process by which an individual plant becomes tolerant to the effects of freezing during a period of weeks to months.
*Paradormancy*: temporary suspension of meristem activity controlled by leaf on apical or axillary buds.
*Pruning*: Removing woody parts (including or sparing reproductive organs) from the canopy.
*Radiative frost*: Frost caused by radiative cooling, i.e., the process by which a body loses heat by thermal radiation, usually infrared.
*Supercooling*: Also known as undercooling, this is when the temperature of a liquid or a gas falls below its freezing point without it becoming solid.
*Thinning*: removing fruits or flowers either singly or in clusters.
